# Mode of Action of Condensed- and Gaseous-Phase Fire Retardation in Some Phosphorus-Modified Polymethyl Methacrylate- and Polystyrene-Based Bulk Polymers

**DOI:** 10.3390/polym13193402

**Published:** 2021-10-03

**Authors:** Paul Joseph, Malavika Arun, Stephen Bigger, Maurice Guerrieri, Doris Pospiech, Christina Harnisch

**Affiliations:** 1 Institute for Sustainable Industries and Liveable Cities, Victoria University, P.O. Box 14428, Melbourne, VIC 8001, Australia; malavika.arun@vu.edu.au (M.A.); Stephen.Bigger@vu.edu.au (S.B.); Maurice.Guerrieri@vu.edu.au (M.G.); 2 Leibniz-Institute of Polymer Research Dresden, Hohe Straße 6, 01069 Dresden, Germany; pospiech@ipfdd.de (D.P.); Harnisch@ipfdd.de (C.H.)

**Keywords:** polymethyl methacrylate, polystyrene, phosphorus modification, char analysis, vapour-phase inhibition, mode of action of fire retardance

## Abstract

The aspects of fire retardation in some phosphorus-modified polymethyl methacrylate (PMMA) and polystyrene (PSt) polymers are reported in the present paper. Both *additive* and *reactive* strategies were employed to obtain the desired level of loading of the phosphorus-bearing compound/moiety (2 wt.% of P in each case). Test samples were obtained using bulk polymerization. The modifying compounds contained the P-atom in various chemical environments, as well as in an oxidation state of either III or V. With a view to gain an understanding of the chemical constitution of the gaseous products formed from the thermal decomposition of liquid additives/reactives, these materials were subjected to GC/MS analysis, whereas the decomposition of solid additives was detailed using the pyrolysis-GC/MS technique. Other investigations included the use of: Inductively-coupled Plasma/Optical Emission Spectroscopy (ICP/OES), solid-state NMR and FT-IR spectroscopy. In the case of PMMA-based systems, it was found that the modifying phosphonate ester function, upon thermal cracking, produced ‘phosphorus’ acid species which initiated the charring process. In the case of solid additives, it is more likely that the resultant phosphorus- and/or oxygenated phosphorus-containing volatiles acted as flame inhibitors in the gaseous phase. With the PSt-based systems, a probable process involving the phosphorylation of the phenyl groups leading to crosslinking and char formation is feasible.

## 1. Introduction

Polymethacrylate (PMMA) and polystyrene (PSt) are frequently used in the construction sector; the former as transparent *Perspex* sheets and the latter as an insulation material. Transparency, ease of processing and relatively good resistance to weathering are the factors that make PMMA a suitable substitute for glass-based construction elements, whereas the inherent thermal properties of PSt, especially in the foamed state (e.g., expanded polystyrene: EPS), means it can function as a good insulating material. However, both materials are highly amenable to thermal degradation, resulting in the release of low-molecular-weight gaseous fragments (including the monomeric species), which in turn can form flammable mixtures with an ambient air. The flammable mixtures formed can easily undergo combustion in the presence of a suitable ignition source or spontaneously at temperatures greater than the ignition temperature, subsequently producing toxic vapours and gases [1].

Fire safety regulations are adopted and strictly enforced in recent times with an aim to protect high-rise buildings especially, where many polymer-based construction components are used. The tendency of polymeric materials to melt and flow, forming a pool of flammable decomposition products, can also constitute a very serious secondary hazard as this can often lead to the further burning of surrounding fuel loads [2]. Generally, this tendency strongly depends on the class of the polymeric material (e.g., thermoplastic, or thermoset) as well as on their chemical constitutions [3]. In addition, common polymers exhibit a wide range of propensities for thermal degradation. Usually, they require a temperature range from approximately 270–470 °C to undergo decomposition to generate volatile fuel fragments which may lead to combustion. However, in the case of foamed products like polystyrene foams, these products are highly ignitable even in the presence of low-intensity sources for piloted ignition [4,5,6,7]. The main reason behind this relatively high ignitability/flammability can be attributed to ease of the monomer liberation at a low temperature [1]. Polymethyl methacrylate (PMMA) is a classic example of a polymer which, upon thermal decomposition, produces a near quantitative yield of the monomer (MMA), through a chain ‘unzipping’ process [8]. Furthermore, owing to its complete decomposition at higher temperatures, PMMA leaves no char residue after a fire. Several incidents of fire hazards are reported, where PMMA melts, and thus enhances the flame spread. During the burning process, polystyrene can also melt, flow and drip, which can lead to a distributed fuel load feeding into an enhanced flame spread [2]. 

Attempts to fire retard both PMMA and PSt are well documented. For this purpose, it is often necessary to treat the parent polymeric matrix through a suitable methodology, in which an appropriate combustion inhibitory reagent (e.g., a flame retardant, FR) is incorporated into the final product. Generally, a large number of flame retardants were used for many years to protect polymeric materials from decomposition and subsequent combustion [9]. These flame retardants may be mixed as additives in the polymer matrix by physical means. Another way of improving flame retardancy is to prepare inherently less flammable polymers through the copolymerization with compounds that can impart fire resistance [10,11]. Organohalogen compounds are primarily used for this purpose as they are excellent in reducing the flammability of polymers. However, the environmental toxicity of these materials caused a ban on their use for commercial purposes. Therefore, inorganic fillers such as magnesium hydroxide, graphene oxide and modified nanoparticles such as clays and silica, etc., and phosphorus-containing compounds are now more commonly used [11]. The efficiency of phosphorus-based compounds generally depends on several factors: the chemical environment and oxidation state of the P atom, volatility, and the nature of the decomposition products formed upon thermolysis, etc. [12]. The condensed-phase activity of phosphorus compounds predominantly involves char formation which is facilitated by the dehydration of the polymeric structure leading to cyclization, cross-linking and aromatization/graphitization [13]. Cross-linking can be also induced by the decomposition by-products of the phosphorus compounds. For polymers with hydroxyl or amino, and groups in their monomeric units, such as in the case of cellulose or wool, phosphorus compounds work mainly in the condensed phase. In the case of olefin-based polymers, these compounds act mainly in the gaseous phase by scavenging radicals such as H*, HO* and preventing their oxidation. Phosphorus-based compounds, such as phosphine, phosphine oxides, phosphonates, and phsophorylamino esters are found to have similar effects to halogen-containing compounds when incorporated into PMMA and PSt [12,14,15].

Deciphering the processes that are responsible for the flame retardant effects of some phosphorus-modified PMMA- and PSt-based bulk polymer samples is attempted by using a variety of analytical techniques. The syntheses of the control and phosphorus-modified PMMA and PSt systems, their spectral characterization, and thermal and calorimetric investigations are communicated separately [16,17]. The analytical investigations primarily included char analyses (using Inductively-coupled Plasma/Optical Emission Spectroscopy: ICP/OES; solid-state NMR spectroscopy: ^13^C and ^31^P; FT-IR, in Attenuated total reflectance mode-ATR), and investigation of gaseous-phase products using GC/MS and pyrolysis GC-MS measurements). 

## 2. Materials and Methods

All the chemicals, reagents and solvents were purchased from Aldrich Chemical Company, except the following: 9,10-dihydro-9-oxa-10-phosphaphenanthrene 10-oxide (DOPO) and diethyl-1-propylphosphonate (Thermofisher Scientific). Generally, the solid compounds were used as received, whereas liquid reagents and solvents were, optionally, dried by keeping them over molecular sieves (4 Å). Furthermore, thermally labile initiators and monomers were stored under sub-ambient temperatures in a refrigerator, or in a freezer, as the case may be. The inhibitors (typically hindered phenolic compounds, such as hydroquinone monomethyl ether), were removed from methyl methacrylate by passing through proprietary inhibitor removal columns, purchased from Aldrich Chemical Company.

The synthetic procedures for the preparation of the precursor compounds, the additive (diethylbenzylphosphonate), and various functional monomers and polymeric products are given elsewhere [16,17]. These additives/reactives included: triphenylphosphine (TPP); triphenylphosphineoxide (TPPO); 9,10-Dihydro-9-oxa-10-phosphaphenenthrene-10-oxide (DOPO); diethylphosphite (DEHPi); triethylphosphite (TEPi); triethylphosphate(TEPa); diethylpropylphosphonate (DEPP); diethylbenzylphosphonate (DEBP); diethyl-1-(acryloyloxyethyl)phosphonate (DE-1-AEP); acrylic acid-2-[(diethoxyphosphoryl) methyl amino] ester (ADEPMAE); diethyl-2-(acryloyloxy)ethylphosphate (DEAEPa); diethyl-p-vinylbenzylphosphonate (DEpVBP) (see Table 1 below).

All the polymeric products, including their controls, were synthesized through the bulk polymerization route. In this method, the required amount of monomer(s) and initiators were stirred thoroughly in a conical flask under a nitrogen atmosphere for the specified duration (*ca*. 1 h at 70–80 °C for MMA and ca. 5 h at 70 °C for St), until a visible increase in the viscosity was observed. The calculated amount of the additive/reactive was then added, and stirred for another 1 h, and the mixture was subsequently poured into an aluminium pan of ca. 50 mL volume and the pan was stoppered with an aluminium lid. The pan was placed in an air oven preheated at 40 °C and kept for curing for about 20 h. During the second stage of curing, the temperature of the oven was raised to 60 °C for 8 h. In the case of the PMMA-based polymers, another 20 h of curing at 80 °C was conducted, whereas for St-based polymers the corresponding stage involved 20 h of curing at 80 °C followed by a period of 3 h at 100 °C. The final products were extracted from the pans after cooling to room temperature. 

The detailed procedures for sample preparation, instruments used, operating parameters, and data accusation and processing, relating the various analytical instrumentation and associated techniques were published previously [18]. These involved the following: the phosphorus contents of aqueous extracts of the test samples were measured in triplicate by using Shimadzu ICPE-9000, and the average values were taken. The solid-state NMR (^31^P with CP/MAS mode) spectra of the char residues was obtained by employing a 500 MHz Bruker machine at ambient probe conditions, typically at 10 kHz rotor speed, and the signals were calibrated against phosphoric acid as the external calibrant. The raw data were then processed by using a proprietary software from the manufacturer (TopSpin 4.0.6). A For FT-IR measurements, a Perkin-Elmer 1600 model instrument was used, in which infrared radiation in the range 4000 to 600 cm^−1^ was absorbed by the test sample in the attenuated total reflectance (ATR) mode (typically 32 scans at a resolution of 4 cm^−1^).

Pyrolysis-GC/MS was performed with the pyrolysator Pyroprobe 5000 (CDS Analytical, Inc., Oxford, PA, USA) with platinum filament coupled with gas chromatograph GC7890A (Agilent Technologies, Santa Clara, CA, USA), and with GC column HP-5MS (non-polar, length: 30 m; inner diameter: 250 μm; layer thickness: 0.25 μm), (Agilent Technologies, Santa Clara, CA, USA). The carrier gas was helium with a gas flow rate of 1 cm^3^ min^−1^. The GC was equipped with the mass-selective detector MSD 5975C inert XL EI/CI (Agilent Technologies, Santa Clara, CA, USA) with a mass scan range between 15–550 m/z and EI at 70 eV. The samples were pyrolyzed at the temperatures of maximum mass losses found in TGA. The inlet temperature of the GC was variable, and the oven temperature programme was fixed (2 min at 50 °C; heating with 12 K min^−1^ to 280 °C). Here, the temperature for the pyrolysis of the admixtures with PMMA and PSt with the solid additives (TTP, TPPO and DOPO) were selected from the first derivative of the corresponding TGA runs obtained at a heating rate of 10 °C min^−1^ in nitrogen (PMMA: 381 °C; PMMA + TPP: 246 °C; PMMA + TPPO: 371 °C; PMMA + DOPO: 388 °C; PSt: 412 °C; PSt + TPP: 418 °C; PSt + TPPO: 416 °C; PSt + DOPO: 431 °C).

## 3. Results

In the following sections, some possible components of processes operating in the condensed and gaseous phases of the PMMA- and PSt-based systems are given. These were primarily inferred from the results obtained through a combination of analytical techniques (ICP/OES, solid-state NMR and FT-IR) and, optionally, from some hyphenated methods (GC/MS and pyrolysis-GC/MS), the latter being mainly used for gaseous phase analyses. The modes of action for PMMA- and PSt-based materials are given separately.

### 3.1. Mode of Action of PMMA-Based Materials

#### 3.1.1. Phosphorus Contents of the Char Residues (ICP/OES)

The phosphorus contents of the char residues were determined through ICP/OES measurements. The reported values in Table 2 are averages over triplicate runs for each sample. In order to obtain enough char residues for the analyses (ICP/OES and solid-state NMR), smaller pieces of the samples sourced from the plaques were taken in 50 mL porcelain crucibles, and then made to undergo a forced flaming combustion inside a fume cupboard, using a butane troch that had a flame length of ca. 4 cm. It should be noted that since the combustibility of the samples differed widely, the duration of the application of the pilot needed to be varied accordingly. Hence, no meaningful assessments of the char yields were made, as this essentially required the duration of the impingement of the flame in each case to be exactly the same. Table 2 also includes the ratios of the P-loading in the unburnt samples (2 wt.%) to P-contents in char residues (in wt.%).

It can be clearly noticed from the above table that several systems retained relatively higher amounts of P in the char residues (e.g., PMMA + DEHPi, PMMA + TEPa, PMMA + DE-1-AEP, PMMA + DEAEPa, PMMA + ADEPMAE and PMMA + DEpVBP), whereas the corresponding values were much lower for the other materials. It is also interesting to note that the system with the P/N-monomer yielded the maximum amount of char (ratio = 17.6). The retention of P in the char residues is indicative to the condensed phase activity of the modifying compounds/groups; however, an unambiguous ranking based on the ratios of the P wt.% in the char residue to those of the unburnt material alone is not possible, especially given that the actual char yield in each case is bound to differ substantially. Nevertheless, the quantitative estimations of P in the char residues can be considered as a useful exercise, as it provides some corroborative evidence to the inferences deduced from their corresponding ^31^P solid-state NMR spectra.

#### 3.1.2. Solid-State NMR

**Figure 1:**
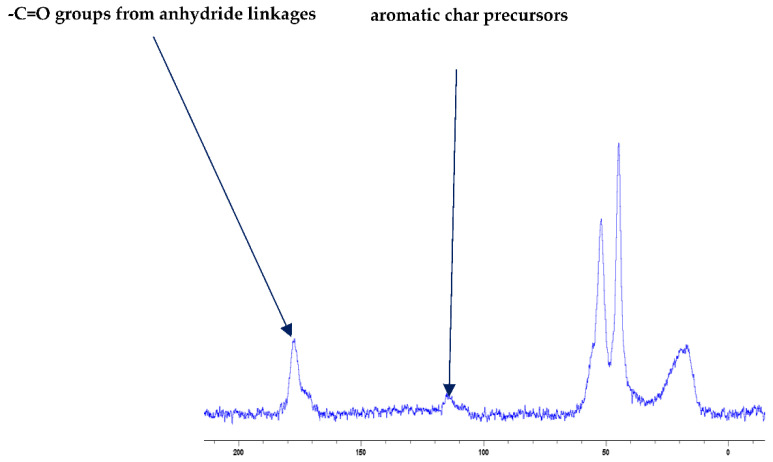
Solid-state ^13^C-NMR spectrum of char obtained from PMMA + DE-1-AEP (the abscissa denotes the chemical shift values, *δ*, in ppm and the ordinate corresponds to the signal intensity in arbitrary units).

**Figure 2:**
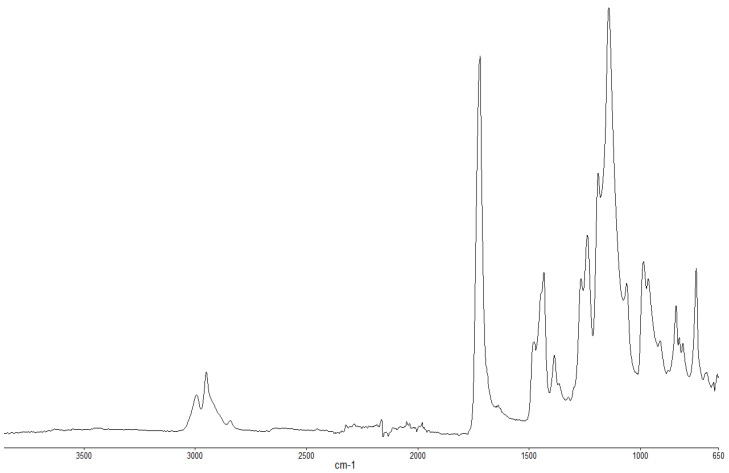
FT-IR spectrum of PMMA (unburnt sample).

**Figure 3:**
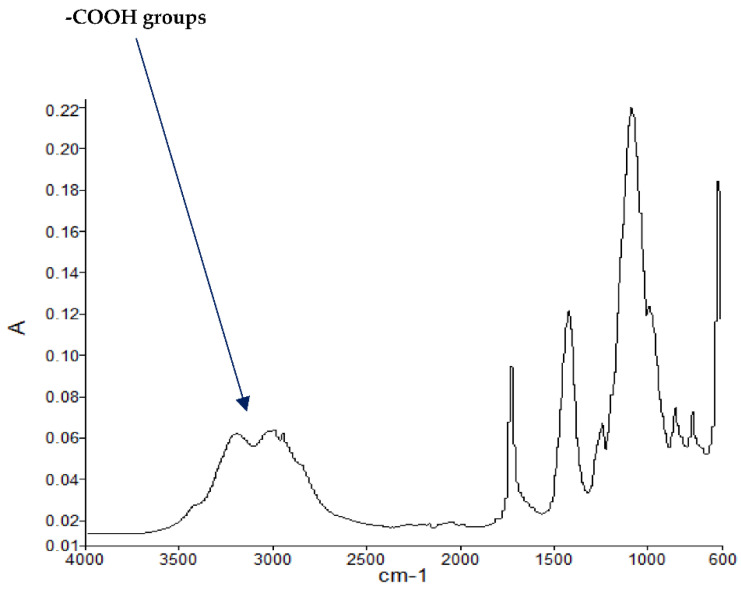
FT-IR spectrum of PMMA + DEpVBP (partially burnt sample).

**Figure 4:**
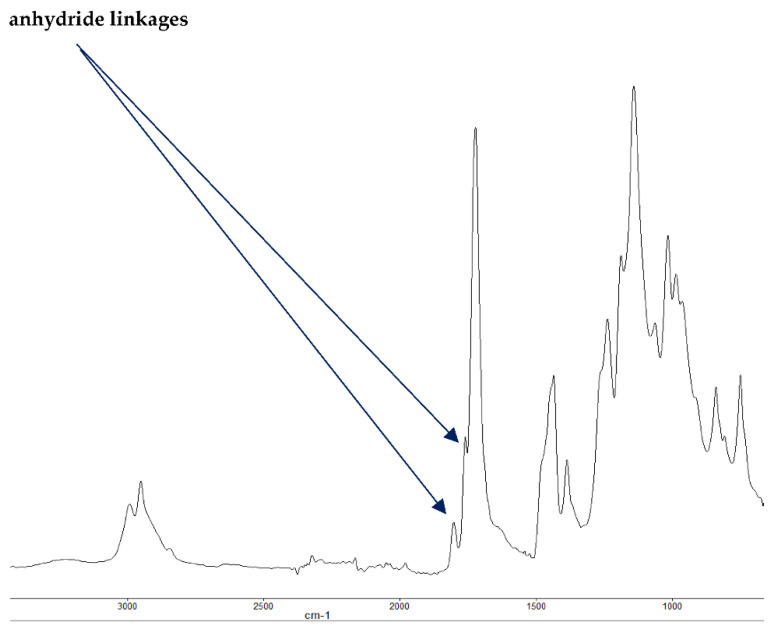
FT-IR spectrum of PMMA + DE-1-AEP (partially burnt sample).

Even though the char residues predominantly consist of carbon atoms, residual protons can also be present to varying degrees depending on the degree of carbonization. Therefore, the scalar coupling patterns owing to the residual protons are found to be present in the ^31^P spectra (as protons are not decoupled during the acquisition of the spectra in the present case), especially in cases where relatively larger amounts of unburnt additives/reactive moieties are present in the test samples (e.g., PMMA + TPPO). Furthermore, the signals in the solid-state NMR spectra are generally less discernible than the corresponding solution-state spectra, primarily owing to dipolar broadening. Therefore, the chemical shift values (e.g., *δ* in ppm, except for the ^13^C spectrum of PMMA + DE-1-AEP: Figure 1) were chosen only. Here, the ^31^P signals from the char residues with those of the additives/reactives recorded in the solution-state spectra were also compared to the corresponding solid-state values obtained, where a broad-band decoupling of protons was used. In this context, it was assumed that the chemical shift values of specific ^31^P signals did not differ largely when recorded in both states (see Table 3). Such an assumption was validated in the case of the solid additives (e.g., TPP, TPPO and DOPO), by recording the spectra through both techniques and comparing the chemical shift values. In the following section, the ^31^P spectra and optionally, the ^13^C and FT-IR spectra of chars obtained from the PMMA-based materials are given (Figure 1, Figure 2, Figure 3 and Figure 4).

**Figure 5:**
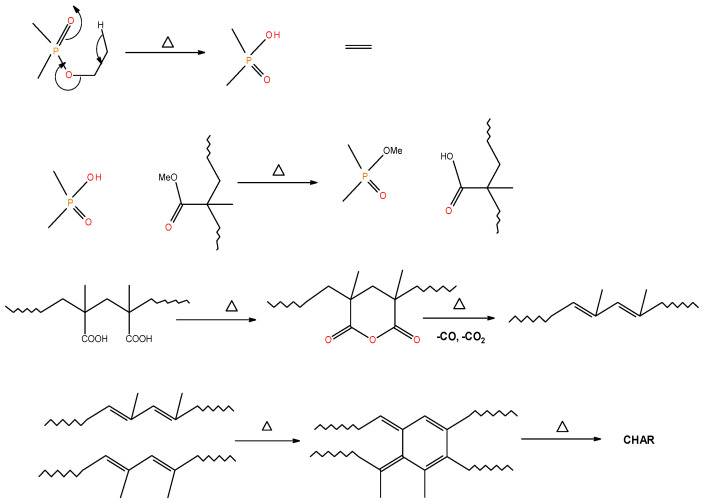
A schematic representation of the formation of char residues in the PMMA-based polymers upon flaming combustion [19].

**Figure 6:**
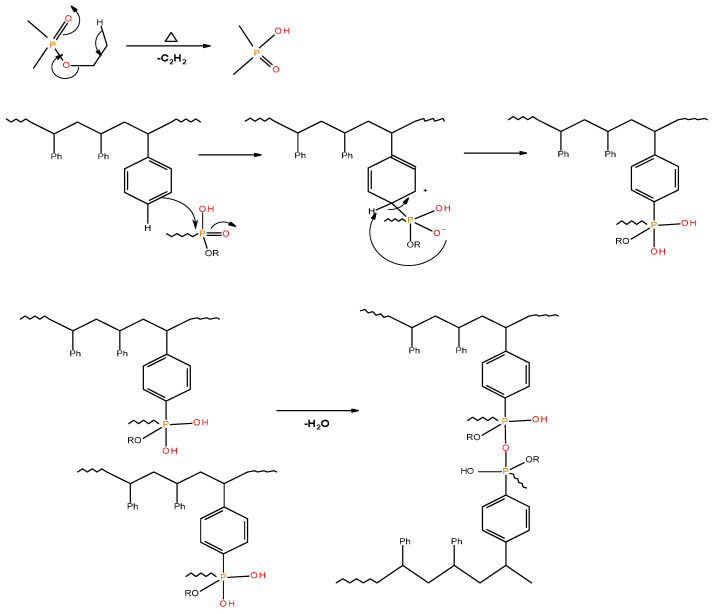
A schematic representation of the possible pathway leading to cross-linking and formation of polyphosphate linkages in modified PSt-based materials.

Generally, the presence of ^31^P signals around ∂ = 0 ppm can be considered to arise from ‘phosphorus’ acid species from the thermal cracking of the P-bearing additives/groups [19]. The formation of such acidic species is especially feasible in the case of phosphites, phosphates, and phosphonate/phosphorlamino esters (see Figure 5). The ‘phosphorus’ acid species thus formed can condense to form polyphosphoric acid species, where the ^31^P signal shifts slightly to the negative region. Such condensation reactions can also result in the cross-linking of chains, as shown to occur in the case of PSt-based polymers (see also Figure 6). Generally, the sharper signals nearer to 0 ppm can be clearly assigned to ‘free’ (e.g., unbound) phosphoric acid. In both cases, this can lead to a broadening of the signal when compared to the signals arising from more readily mobile ‘phosphorus’ acid species which remain in the char structure as unattached entities (see, for example, in the case of PMMA + ADEPMAE).

It is also reported in the literature that, in the case of PMMA-based systems, the ‘phosphorus’ acid species further trans-esterify the ester groups in the PMMA chain leading to pendent carboxylic acid functions which, under the influence of heat, subsequently form anhydride links [19]. Once formed, the anhydride links can undergo decarboxylation reactions (see in Figure 1) and Diels-Alder type additions, etc., eventually resulting in precursors for carbonaceous char (see also in the FT-IR spectrum given in Figure 4). In the case of the aromatic phosphine/phosphine oxide, it is more likely that the ^31^P signal originates from the unburnt additives and, in some instances, the signal pattern also seems to incorporate the^1^H-^31^P couplings. With the limited amount of information in hand from the solid-state NMR spectra, and through complementary inferences made from the results of the hyphenated technique (pyrolysis-GC/MS), the shift of the signals towards the range of ∂ ∼ 0- 2 ppm in the cases of TPP, TPPO and DOPO cannot be easily explained.

#### 3.1.3. FT-IR Spectroscopy

The FT-IR spectra of ‘partially’ burnt materials from PMMA (by igniting the tip of a solid plaque) and some optional modified systems recorded in the ATR mode are shown in Figure 2, Figure 3, Figure 4. The formation of carboxylic acid groups and anhydride linkages can be clearly seen in the spectra of the ‘partially’ burnt residues from the modified systems, whereas such features are absent in the spectrum obtained from the virgin material. The complementary information regarding the formation of the anhydride linkages and the formation of aromatic species is also found in the ^13^C NMR spectra of the char residues (as shown in Figure 1).

#### 3.1.4. Pyrolysis-GC/MS of PMMA-Based Materials

In the following sections, the pyrolysis-GC/MS of the unmodified PMMA and the modified versions containing the solid additives are provided. Here, the temperature for pyrolysis was selected from the first derivative of the TGA run obtained at a heating rate of 10 °C min^−1^ [16]. The major component (e.g., MMA monomer) appeared with a retention time of 2.83 min, and other minor components originating from the initiators (benzoylperoxide: BPO and dicumylperoxide: DCP) in varying amounts can be observed as follows: 7.69 min (acetophenone); 8.04 (methylcumyl ether); 14.48 min (phenylbenzoic acid); 16.72 min (methylhexadecanoate); 18.31 min (methyloctadecanoate), etc.

In the case of the PMMA + TPP system, apart from the major components (MMA monomer from the homopolymer at 2.99 min and the additive, TPP, at 18.77 min), several smaller fragments from the initiator species were also observed: 6.52 min (methyl styrene); 7.71 min (acetophenone); 7.95 (*α*-cumyl alcohol); 8.10 min (methylbenzoate). It appears that during the pyrolysis at 246 °C, the base matrix and additive degrade independently without any noticeable interaction. The same type of behaviour was observed in the case of the other two modified PMMA systems (PMMA + TPPO and PMMA + DOPO. Generally, some corroborative evidence regarding the mode of action of the modifying groups can be also drawn from the corresponding thermo-gravimetric analysis (TGA), differential scanning calorimetry (DSC) and pyrolysis combustion flow calorimeter (PCFC) tests [16].

### 3.2. PSt-Based Materials

#### 3.2.1. Phosphorus Contents of Char Residues (ICP/OES)

The ratio of the phosphorus content in the unburnt material and the corresponding retention in the char residue can be considered as an indication of the extent of condensed phase activity of the modifying agent (see in Table 4). Enhanced degrees of P-retention were observed for several PSt-based systems, such as PSt with TPP, TPPO, ADEPMAE, DEAEPa and DEpVBP. As in the case of the PMMA-based systems, the maximum phosphorus retention was observed for PSt + ADEPMAE. Here, the conditions under which the char residues were obtained also varied in each case in terms of the duration of the pilot applied. Hence, any such general inferences that can be drawn in this context, judging by the P-loadings alone, need to be treated with caution. 

#### 3.2.2. Solid-State NMR

As in the case of the ^31^P NMR spectra of char residues from the PMMA-based systems, the appearance of signals around 1 ppm cannot be explained easily in the corresponding spectra for char residues from PSt-based systems containing solid additives (TPP and TPPO) (see in Table 5). It is highly unlikely that these signals arise due to the presence of ‘phosphorus’ acid species from the additives, as the breakage of C–P bonds yields resonance-stabilised phenyl species. This will, in turn, facilitate the release of P. and PO. radicals into the vapour space. The corroborative evidence of this type of bond cleavage can be drawn from the pyrolysis-GC/MS studies of the solid additives [18]. However, thermal cracking of the ethyl groups (in phosphites, phosphates, and phosphonate/phosphorlaminoester) is quite feasible in producing the corresponding ‘phosphorus’ acid species with ∂ ∼ 0.0 ppm [19]. The ‘phosphorus’ acid species, once formed, can then proceed to phosphorylate the phenyl rings of PSt, and can subsequently result in the cross-linking of the polymeric chains through the condensation of the phosphorylated rings (Figure 6).

#### 3.2.3. Pyrolysis-GC/MS of PSt-Based Materials

Here, the temperature for pyrolysis was selected from the first derivative of the TGA run obtained at a heating rate of 10 °C min^−1^ [17]. As expected, apart from the monomeric units, there were varying amounts of decomposition products from main chain scissions, or from side groups, such as PSt. Furthermore, a small number of minor components emanating from the pyrolysis of the initiator species can also be seen in the chromatogram/mass spectrum (see also Table 6). In the modified versions (PSt + TPP, PSt + TPPO and PSt + DOPO), in addition to the abovementioned fragments, the corresponding additive compounds are found: TPP (18.85 min), TPPO (21.34 min), and DOPO (18.81 min). Once again, no co-operative interaction can be noticed for these solid additives; therefore, it is to be assumed that the homopolymer matrix and the additive compound underwent decomposition independently. It should be noted here that the detection of phosphorus-containing species in the gas phase does not necessarily establish gas-phase activity [20,21].

## 4. Conclusions

In the case of the PMMA-based systems, there is evidence that P-bearing compounds/groups, except TPP, TPPO and DOPO, upon thermal cracking during the early stages of flaming combustion, produce ‘phosphorus’ acid species. These acidic species can subsequently initiate the chemical pathway used to produce char precursors. In the case of TPP, TPPO and DOPO, it is more likely that they produce phosphorus- and/or oxygenated phosphorus-containing volatiles that can act in the gaseous-phase [18]. 

However, in the case of programmed heating under controlled environments, such as in TGA, DSC, PCFC and ‘bomb’ calorimetry, where a flaming mode of combustion is not attained, any such cooperative interaction between the parent polymer matrix and the modifying compound/groups can be assumed to be absent [16]. On the other hand, with PSt-based systems, the modifying moieties seem to exert some degree of cooperative interactions even in the controlled decomposition tests (e.g., TGA and DSC), and certainly in measurements where combustion occurs (PCFC and ‘bomb’ calorimetry) [17]. Here, a probable process involving the phosphorylation of the phenyl rings leading to crosslinking and char formation is proposed. Furthermore, the thermal degradation of polystyrene is strongly dependent on temperature, where the initial event is established to be the rapid evolution of the monomer [22].

## Figures and Tables

**Table 1:** Various additives and reactives used for the bulk polymerization of MMA and St.

Sl. No.	Additive/Reactive	Structure/Oxidation State
1.	Triphenylphosphine (TPP), *additive*	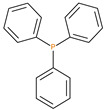
2.	Triphenylphosphineoxide (TPPO), *additive*	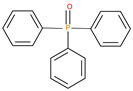
3.	9,10-Dihydro-9-oxa-10-phosphaphenenthrene-10-oxide (DOPO), *additive*	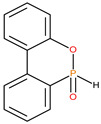
4.	Diethylphosphite (DEHPi), *additive*	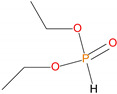
5.	Triethylphosphite (TEPi), *additive*	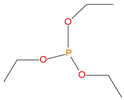
6.	Triethylphosphate (TEPa), *additive*	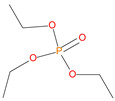
7.	Diethylpropylphosphonate (DEPP), *additive*	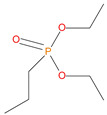
8.	Benzylphosphonate (DEBP), *additive*	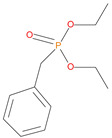
9.	Acrylic phosphonate (DE-1-AEP), *reactive*	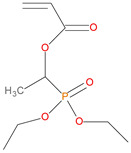
10.	P/N (ADEPMAE), *reactive*	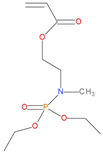
11.	Acrylic phosphate (DEAEPa), *reactive*	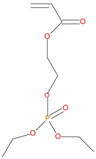
12.	Vinylbenzylphosphonate (DEpVBP), *reactive*	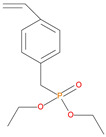

**Table 2:** Phosphorus contents of the unburned samples and char residues of PMMA-based materials.

Sl.No.	Samples	wt.% of P in the Unburnt Sample	wt.% P in Char	Ratio of P in Char to P in Sample
1	PMMA	0	0.00	0.00
2	PMMA + TPP	2	1.97	0.99
3	PMMA + TPPO	2	2.38	1.19
4	PMMA + DOPO	2	5.55	2.78
5	PMMA + DEHPi	2	12.1	6.06
6	PMMA + TEPi	2	3.75	1.88
7	PMMA + TEPa	2	12.2	6.11
8	PMMA + DEPP	2	0.71	0.36
9	PMMA + DEBP	2	7.68	3.84
10	PMMA + DE-1-AEP	2	15.3	7.63
11	PMMA + ADEPMAE	2	35.2	17.6
12	PMMA + DEAEPa	2	9.31	4.66
13	PMMA + DEpVBP	2	10.0	5.01

**Table 3:** The ^31^P chemical shift values (*∂*, in ppm) of the additives/reactives and char residues of PMMA-based materials.

Sl. No.	Additive	∂ Value for ^31^P of the Additives ^#^(Solution-State)	∂ Value for ^31^P of the Char Residue- Prominent Signal (Solid-State)
1	TPP	−7.10	−0.3
2	TPPO	27.6	1.6
3	DOPO	16.7	1.3
4	DEHPi	7.30	*---
5	TEPi	7.30	0.0
6	TEPa	−1.00	1.3
7	DEPP	32.2	*---
8	DEBP	26.4	*---
9	DE−1-AEP	21.4	−0.3
10	ADEPMAE	10.4	−1.3
11	DEAEPa	0.50	*---
12	DEpVBP	26.6	0.3

^#^ recorded with broad-band proton decoupling. * not recorded.

**Table 4:** Phosphorus contents of the unburned samples and char residues of PSt-based materials.

Sl. No.	Samples	wt.% of P in the System	wt.% of P in Char	Ratio of P in Char to P in Sample
1	PSt	0	0.00	0.00
2	PSt + TPP	2	7.56	3.78
3	PSt + TPPO	2	6.19	3.10
4	PSt + DOPO	2	1.62	0.81
5	PSt + DEHPi	2	2.28	1.14
6	PSt + TEPi	2	4.64	2.32
7	PSt + TEPa	2	2.32	1.16
8	PSt + DEPP	2	0.34	0.17
9	PSt + DEBP	2	2.17	1.09
10	PSt + DE-1-AEP	2	3.15	1.58
11	PSt + ADEPMAE	2	15.0	7.48
12	PSt + DEAEPa	2	9.96	4.98
13	PSt + DEpVBP	2	5.48	2.74

**Table 5:** Chemical shift values (∂ in ppm) of P nucleus in the additives/reactives and in the char residue obtained from PSt-based systems.

Sl. No.	Additive/Reactive	∂ Value for ^31^P of the Additives ^#^(Solution-State)	∂ Value for ^31^P of the Char Residue- Prominent Signal (Solid-State)
1	TPP	−7.10	1.00
2	TPPO	27.6	0.30
3	DOPO	16.7	*---
4	DEHPi	7.30	0.30
5	sTEPi	7.30	0.00
6	TEPa	−1.00	0.00
7	DEPP	32.2	*---
8	DEBP	26.4	0.70
9	DE−1-AEP	21.4	0.00
10	ADEPMAE	10.4	0.70
11	DEAEPa	0.50	*---
12	DEpVBP	26.6	−0.30

^#^ recorded with broad-band proton decoupling. * not recorded.

**Table 6:** The retention times and corresponding masses of the major fragments for PSt.

Sl. No.	Retention Time (min)	m/z	Species
1	5.18	104	Styrene
2	6.19	106	Benzaldehyde
3	7.70	120	Acetophenone
4	15.1	208	Styrene dimer
